# Baduanjin improves motor function and cortical activation in children with developmental coordination disorder: a randomized controlled trial

**DOI:** 10.1186/s12906-026-05509-w

**Published:** 2026-08-13

**Authors:** Jiaxin Gao, Xiaohua Ke, Cai Jiang, Hongfei Ren, Jiaqi Wang, Dunbing Huang, Shanli Yang, Zhenhua Wu, Zhonghua Lin, Wei Song

**Affiliations:** 1 Rehabilitation medicine center, Fuzhou University Affiliated Provincial Hospital, Fuzhou, China; 2 Department of Rehabilitation Medicine, Shanghai Fourth People’s Hospital, School of Medicine, Tongji University, Shanghai, China; 3 Encephalopathy and Rehabilitation Center, The Second Affiliated Hospital of Zhejiang Chinese Medical University, Hangzhou, China; 4 Fujian University of Traditional Chinese Medicine Subsidiary Rehabilitation Hospital, Fuzhou, China

**Keywords:** Developmental Coordination Disorder, Baduanjin, Motor Function, Functional Near-Infrared Spectroscopy

## Abstract

**Background:**

Children with developmental coordination disorder (DCD) exhibit significant deficits in both gross and fine motor functions. Baduanjin, a traditional Chinese mind-body exercise, has demonstrated potential in improving motor functions, including balance and neuromuscular coordination, in various rehabilitation settings. This study aims to investigate the effects of Baduanjin intervention on motor function in children with DCD, and further explore whether its potential benefits are mediated through improved cortical activation in motor-related brain regions, as reflected by cerebral hemodynamic responses.

**Methods:**

This single-blind randomized controlled trial enrolled 40 children aged 5–10 years who met the DSM-5 diagnostic criteria for DCD and had an MABC-2 standard score of ≤ 7. Participants were randomly assigned, using a SAS 9.4-generated randomization sequence with sealed opaque envelopes for allocation concealment, to either an experimental group receiving Baduanjin training plus health education or a control group receiving health education alone. The intervention lasted 8 weeks, with five sessions per week, including two instructor-led hospital sessions and three parent-supervised home sessions. After the intervention, motor coordination and balance were assessed using the Movement Assessment Battery for Children-2 (MABC-2) and the ProKin 254 balance test system, respectively. In addition, functional near-infrared spectroscopy (fNIRS) was used to measure hemodynamic responses in motor-related cortical regions during functional tasks, to evaluate cortical activation and examine its associations with changes in motor function.

**Results:**

The per-protocol (PP) dataset included 36 participants, whereas the intention-to-treat (ITT) dataset included all 40 randomized children. Both PP and ITT analyses showed that the experimental group had significantly higher total MABC-2 scores than the control group (PP: *P* = 0.002; ITT: *P* = 0.001). Subscale analyses demonstrated significant improvements in aiming and catching (PP: *P* = 0.019; ITT: *P* = 0.005) and balance (PP: *P* = 0.008; ITT: *P* = 0.004). In addition, the experimental group showed significant improvements in key static balance parameters, including reduced sway area (PP and ITT: *P* < 0.001) and shorter sway path length (PP and ITT: *P* < 0.001). Dynamic postural stability also improved significantly (PP and ITT: *P* = 0.004). However, no statistically significant between-group differences were observed in manual dexterity (PP and ITT: *P* > 0.05). fNIRS results revealed that during the aiming and catching task, the experimental group exhibited significantly greater activation in the left premotor cortex and supplementary motor area (PMC + SMA) compared to the control group (CH31: FDR-adjusted *P* = 0.015; CH32: FDR-adjusted *P* = 0.041). In the one-leg balance task, the experimental group showed significantly enhanced activation in the left hemispheric regions, including the frontopolar area (FPA), dorsolateral prefrontal cortex (DLPFC), inferior frontal gyrus (IFG), primary motor cortex (M1), as well as the PMC + SMA (FDR-adjusted *P* = 0.018). No significant between-group differences in brain activation were observed during the manual dexterity task (FDR-adjusted *P* ≥ 0.05). Furthermore, correlation analysis indicated a significant positive association between improvements in motor performance and activation of motor-related brain regions in the experimental group.

**Conclusions:**

Baduanjin training improved motor coordination and balance in children with DCD. The observed changes in activation within motor-related cortical regions provide preliminary support for a possible neurophysiological basis of these functional improvements, although the underlying mechanisms require further investigation.

**Trial registration:**

ChiCTR, ChiCTR2300078980. Registered 22 December 2023, https//www.chictr.org.cn. The study protocol has been published (doi 10.1136/bmjopen2024084061).

**Supplementary Information:**

The online version contains supplementary material available at https://doi.org/10.1186/s12906-026-05509-w.

## Backgroud

Developmental coordination disorder (DCD) is a prevalent neurodevelopmental disorder in children [1], primarily characterized by significant difficulties in motor coordination and skill acquisition [2], with an estimated prevalence of 5%-6% [3, 4]. Children with DCD commonly display motor impairments across multiple domains, such as poor postural control, limited balance ability, and inefficient sensorimotor integration during task performance [5, 6]. These motor impairments are often accompanied by broader neurocognitive dysfunction, including immature motor control strategies, limited motor learning capabilities, and deficiency in executive function and attentional modulation [4]. The motor skill deficits substantially compromise children’s autonomy and independence in daily activities [7–9], and may further interfere with academic skill development, particularly in reading, writing, and participation in physical education [9, 10], ultimately adversely affecting academic performance and educational attainment [11]. Early diagnosis and timely intervention are therefore essential to improve motor proficiency in children with DCD [7, 12, 13].

Currently, motor-based interventions remains the primary intervention strategy for children with DCD [4, 12, 14]. Our previous systematic review and meta-analysis demonstrated that motor-based interventions significantly enhance overall motor skills, body function, and activity performance in children with DCD [14]. Baduanjin, a traditional Chinese mind-body exercise rooted in Chinese medicine [15, 16], has been extensively studied for its therapeutic benefits. Accumulating evidence indicates that regular Baduanjin practice can markedly improve motor functions, particularly balance and neuromuscular coordination [17–19]. Baduanjin is characterized by gentle, slow-paced movements with coordinated trunk and limb control [20]. Its practice involves dynamic postural adjustments, such as weight shifting and heel lifting, which require the integration of balance, flexibility, and neuromuscular control, and may therefore help enhance motor system function. Jiang et al. revealed that Baduanjin training elicits significantly greater displacement amplitude, velocity, and acceleration of center-of-pressure trajectories compared to walking, establishing its efficacy as a balance-enhancing modality [21]. Supporting this, Yuen et al. demonstrated in chronic stroke patients that an 8-week Baduanjin program yielded superior improvements in balance and mobility compared to conventional fitness training [22]. Although these findings were derived from populations other than children with DCD, they provide preliminary support for the feasibility of Baduanjin as a motor-oriented intervention. However, whether similar functional benefits can be achieved in children with DCD remains unclear.

In addition, several studies have suggested that Baduanjin training may influence cortical function [23, 24]. Yao et al. found that Baduanjin training significantly increased oxygenated hemoglobin concentration in the left dorsolateral prefrontal cortex (DLPFC) and right inferior frontal gyrus of elderly adults [23]. Yuan et al. demonstrated that Baduanjin training markedly activated brain regions associated with sensorimotor function, including the left postcentral gyrus, in older adults [24]. Taken together, these findings suggest that Baduanjin may improve motor function by affecting cortical activity related to motor control. Children with DCD, however, exhibit atypical cortical activation patterns compared with their typically developing peers. Neuroimaging studies have revealed both structural and functional abnormalities in brain networks involved in motor information processing and motor control in this population [25–27]. Therefore, it is reasonable to hypothesize that Baduanjin training may improve motor function in children with DCD by enhancing activation in motor-related cortical areas. Motor-related cortical regions relevant to DCD include the primary motor cortex (PMC/M1) [28, 29], premotor cortex and supplementary motor area (PMC + SMA/M2) [30], primary somatosensory cortex (S1) [31–34], secondary somatosensory cortex (S2) [35–37], and prefrontal cortex (PFC) [31, 38, 39], which together contribute to motor planning, execution, sensory feedback integration, and cognitive regulation of movement. Investigating whether Baduanjin influences activation in these regions may help clarify the neurophysiological basis of its potential effects on motor function in children with DCD.

Commonly used neuroimaging techniques include magnetic resonance imaging (MRI), electroencephalography (EEG), and functional near-infrared spectroscopy (fNIRS). MRI requires strict restriction of body movement during scanning, and EEG is highly susceptible to motion artifacts during data acquisition. In contrast, fNIRS demonstrates strong resistance to motion-related artifacts, making it particularly well-suited for evaluating brain function during tasks that involve a certain degree of movement [40]. fNIRS is a non-invasive and portable technique that quantifies cerebral hemodynamics in the cortical regions, offering both high temporal and spatial resolution for time-sensitive assessment of brain function [41]. This technique indirectly reflects cortical activation by continuously monitoring changes in the concentrations of oxygenated hemoglobin (HbO) and deoxygenated hemoglobin (HbR) in the cortex during task performance [42, 43]. Several previous studies have employed fNIRS to investigate the neurophysiological mechanisms in children with DCD [44, 45].

Therefore, this study focuses on children with DCD and employs a randomized controlled trial design to evaluate the effectiveness of a Baduanjin training for children who meet the Diagnostic and Statistical Manual of Mental Disorders, Fifth Edition (DSM-V) diagnostic criteria for DCD. Motor coordination is designated as the primary outcome, with balance ability as a secondary outcome, to assess the impact of Baduanjin training on motor function in this population. In parallel, fNIRS is used to collect hemodynamic data from motor-related cortical regions during functional tasks. The study aims to determine whether the effects of Baduanjin are mediated by enhanced cortical activation, thereby exploring the potential cerebral oxygenation mechanisms underlying its therapeutic efficacy in children with DCD.

## Methods

### Study design

This study employed a randomized, parallel-controlled trial design, with bias minimized through the use of a single-blind method. The trial has been registered with the Chinese Clinical Trial Registry (trial registration number: ChiCTR2300078980), and the study protocol has been published (see Gao et al. [46]). Ethical approval was obtained from the Medical Ethics Committee of Shanghai Fourth People’s Hospital, affiliated with Tongji University (Approval No. 2023080-001). The reporting for the trial followed the Consolidated Standards of Reporting Trials (CONSORT) guideline [47].

### Recruitment procedure

Participant recruitment was conducted through the Departments of Rehabilitation Medicine and Pediatrics of Shanghai Fourth People’s Hospital. According to the published study protocol [46], recruitment advertisements targeting children with DCD were disseminated through the official platforms of Shanghai Fourth People’s Hospital and its Department of Rehabilitation Medicine, and printed materials were displayed in the pediatric outpatient area. Parents of potential participants initiated the screening process by completing the DCD-Q questionnaire and providing a medical history. Children with unexplained motor skill deficits then underwent further evaluation using the MABC-2 and the Wechsler Intelligence Scale for Children to confirm eligibility. Eligible families were approached by clinical team members either during outpatient visits or by telephone and were invited to provide informed consent. The study period was from 1 September 2023 to 1 March 2025. In total, 48 children were screened before enrollment, of whom 8 were excluded for not meeting the inclusion criteria, resulting in 40 eligible participants who were randomized.

### Participants

The trial was conducted in strict accordance with the inclusion and exclusion criteria outlined in the study protocol [46]. Children aged 5 to 10 years who met the DSM-V diagnostic criteria for DCD were enrolled [48]. Motor performance was assessed using the Movement Assessment Battery for Children–Second Edition (MABC-2) [49]. In line with the recommendation 12 of the 2019 International Clinical Practice Guideline for DCD, a standard score of ≤ 7 on the MABC-2 (i.e., a total percentile rank ≤ 16 or a total score ≤ 67) was used as the diagnostic threshold for DCD [4]. All participants were assigned to groups strictly according to the predetermined randomization procedure. The random allocation sequence was generated using SAS 9.4 software by an independent researcher who was not involved in any aspect of the intervention. Allocation concealment was ensured through the use of sequentially numbered, sealed opaque envelopes. Recruitment staff and intervention implementers operated independently, and both outcome assessors and statistical analysts were blinded to group assignments. Because this was a behavioral intervention, participants and intervention implementers could not be blinded; therefore, the study was conducted using a single-blind design in which outcome assessors and statistical analysts were blinded to group allocation.

However, although the originally planned sample size was 60 participants (i.e., 30 per group) as per the initial protocol, the strict diagnostic criteria for DCD resulted in a limited enrollment rate. To ensure data integrity and adherence to research standards, a total of 40 participants (20 per group) were ultimately enrolled and completed the intervention. To assess the statistical power of the study under the actual sample size, a post-hoc power analysis was conducted using G*Power 3.1 software for the primary outcome measure (MABC-2 total score) (See Figure S1). In the per-protocol (PP) analysis, the comparison of MABC-2 total scores between the experimental and control groups yielded a Mann-Whitney U test result of Z = − 3.017 (n₁ = 20, n₂ = 16), corresponding to an estimated effect size of *r* ≈ 0.503 and Cohen’s d ≈ 1.16. These values were entered into G*Power using the following parameters: test type: “Wilcoxon-Mann-Whitney test (two groups)”; effect size d: 1.16; α error probability: 0.05; sample sizes: 20 and 16; tails: two-sided. The calculated statistical power (1–β) was 0.91. In the intention-to-treat (ITT) analysis (Z = − 3.266, n₁ = n₂ = 20), the estimated effect size was *r* ≈ 0.516 and Cohen’s d ≈ 1.21. Inputting these values into G*Power yielded a statistical power (1–β) of 0.95. These results indicate that despite the actual sample size being smaller than originally planned, the current sample still provided high statistical power in both PP and ITT analyses. Therefore, the statistical validity of the primary outcome is reliable, and the study conclusions remain robust.

### Intervention

A total of 40 eligible children diagnosed with DCD were randomly assigned to either the experimental group or the control group. Participants in the control group received only health education sessions, conducted once every two weeks (45 min per session) over an 8-week period, for a total of four sessions. During the study period, they maintained their usual lifestyle without engaging in any structured physical activity or systematic training programs. The health education was delivered by rehabilitation physicians and focused on increasing family awareness of DCD. Topics included the etiology, pathophysiology, clinical manifestations, and home-based management strategies of DCD. Both parents and children participated in the sessions. Children in the experimental group received the same health education and, in addition, participated in a Baduanjin training program. The Baduanjin exercises were based on the national standard “Health Qigong—Baduanjin” issued by the General Administration of Sport of China in 2003 [46], the detailed description of the standardized Baduanjin exercise protocol is presented in Table S1. The intervention followed a staged training protocol, beginning with approximately 10 min of warm-up, followed by 30 min of Baduanjin exercises, and ending with 10 min of cool-down (Table 1). The full intervention lasted 8 weeks, with five training sessions per week, totaling 40 sessions. During the intervention period, participants were required to attend two instructor-led sessions at the hospital each week. The remaining three sessions were completed at home under parental supervision. Parents recorded the home sessions and submitted the videos via the WeChat platform to the research team. Each training session was evaluated using a standardized Baduanjin scoring sheet (See Figure S2). A session was considered effective if the score reached at least 85 out of 100. If the score was below 85, the child was required to repeat the session until it met the training criteria. The 85/100 score was used as a quality-control threshold to ensure movement quality and the accuracy and consistency of home-based practice. On average, 1.12 repetitions were required per session to meet the training criterion. This procedure was intended for quality control rather than additional task-specific training.

Prior to the formal commencement of the trial, instructional training sessions were conducted. Each child received individualized, face-to-face instruction from a research staff member, who demonstrated the Baduanjin movements and provided real-time guidance. Parents were encouraged to participate alongside their children in order to gain a better understanding of the exercise routine and to effectively supervise home-based practice. Upon completion of the instructional session, a separate researcher assessed each child using the standardized Baduanjin scoring sheet to ensure mastery of the key movement components. Only those who achieved a score of 85 or above were permitted to proceed to the formal training phase. To facilitate home practice, parents were provided with a pre-recorded instructional video and a training logbook. Parents were instructed to guide or assist their child during home sessions as needed. Following this preparatory phase, the 8-week intervention period commenced, with training sessions taking place both at home and in the hospital setting. After each training session, participants were required to complete a practice log entry. Research staff conducted weekly reviews of each child’s logbook to monitor compliance with the home training protocol and to ensure adherence to the prescribed exercise schedule.

**Table 1:** Flowchart of the Baduanjin intervention

Warm-up (10 min)——Under the supervision of a rehabilitation therapist, participants performed full-body joint mobilization and stretching exercises to adequately prepare the body for training.
Baduanjin Practice (30 min)——Children followed a standardized Baduanjin instructional video to perform the exercises.
Cool-down (10 min)——Participants stood in an upright position with relaxed muscles and steady breathing, allowing the body to gradually return to a state of natural balance and calm.

### Measurement

All outcome measures were assessed at baseline and again after completion of the 8-week intervention.

#### Main outcome indicators

##### Motor assessment battery for children-second edition (MABC-2)

Children’s motor coordination was assessed using the MABC-2, adapted to Chinese normative data [49]. The MABC-2 comprises three core domains: manual dexterity, aiming and catching, and balance. These domains collectively represent critical dimensions of motor coordination relevant to children’s physical development. The MABC-2 offers a comprehensive framework for evaluating motor coordination across these fundamental components. The MABC-2 includes three age bands, namely 3–6, 7–10, and 11–16 years, with eight task items for each age band. In the present study, only children aged 5–10 years were included. Raw scores obtained from the test were converted into standard scores ranging from 1 to 19, following the standard score conversion tables outlined in the MABC-2 User Manual.

#### Secondary outcome indicators

##### ProKin 254 balance test system

The ProKin 254 Balance Test System (ProKin 254, TecnoBody, Italy) was utilized to assess both static balance and dynamic postural stability. For static balance assessment, the system records the sway path length and the sway area, which represent the total displacement and area covered by the patient’s center of pressure while maintaining a stationary posture. These metrics reflect the degree of sway in the patient’s center of gravity, with larger values indicating reduced static postural stability. Dynamic postural stability is quantified through the stability limit index, which is calculated as the ratio of the actual displacement distance achieved by the patient in all directions to the theoretical maximum distance estimated by the system based on the patient’s anthropometric data (i.e., height and weight). Higher values on this index denote superior dynamic postural control and enhanced balance function.

#### Neuroimaging assessment

##### fNIRS measurement

**Fig. 1:**
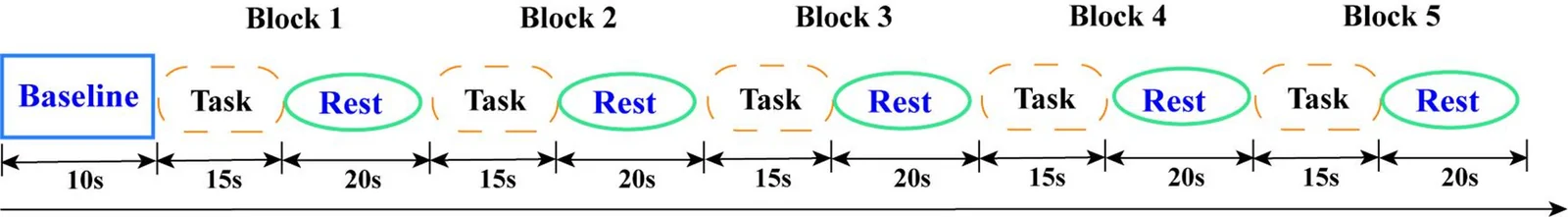
Schematic diagram of the fNIRS task paradigm

**Fig. 2:**
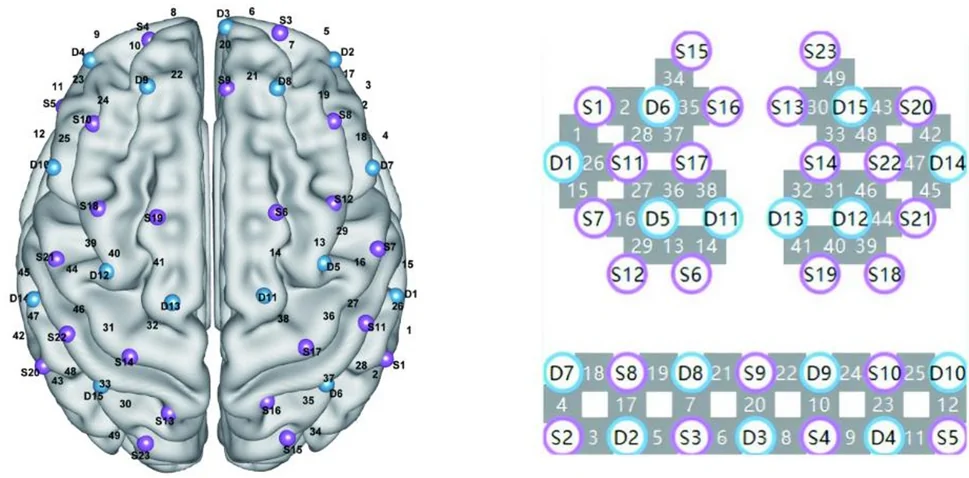
Schematic diagram of fNIRS channels and probe locations

Given that the MABC-2 is a standardized assessment used to comprehensively evaluate various aspects of motor coordination in children, task activities derived from the MABC-2 test were selected to represent motor function during fNIRS measurement for both groups. Due to differences in specific test items for different age groups (3–6 years vs. 7–10 years) within the MABC-2, tasks were standardized by selecting overlapping items from the three main components of the assessment. These included: Drawing Trail (right hand) to represent fine motor skills; Aiming and Catching (Beanbag Throwing) to represent hand-eye coordination; One-Leg Balance (right leg) to represent balance ability. Each of the three selected functional tasks was implemented using a block design. Data were collected for 10 s prior to each task to establish a baseline. Each task evaluation consisted of five blocks, with each block comprising a 15-second task phase followed by a 20-second rest phase (see Fig. 1 for details). Data acquisition was performed using the NirSmart-6000 A fNIRS system (Danyang Huichuang Medical Equipment Co., Ltd., Jiangsu, China). The system utilizes dual-wavelength LED light sources (730 nm and 850 nm) at the emission end and avalanche photodiode detectors at the receiving end. The sampling frequency was set to 11 Hz. The optode arrangement consisted of 23 emitters and 15 detectors, forming 49 effective measurement channels, with inter-optode distances ranging from 2.7 to 3.3 cm (average distance: 3 cm). Regions of interest (ROIs) included bilateral M1, PMC + SMA, S1, S2, PFC (see Fig. 2). fNIRS was used to record hemoglobin concentration changes in these ROIs while children performed the three selected functional tasks.

### Statistical analyses

#### Analytical dataset

In this study, data analysis was conducted using both the PP analysis set and the ITT analysis set. The PP set included participants who strictly adhered to the study protocol, demonstrated good compliance, and completed the case report forms in full; these data were used for statistical analysis. The ITT set included all 40 randomized children, who were analyzed according to their original group assignment, regardless of adherence to the intervention. Missing outcome data were handled using multiple imputation by chained equations under the missing-at-random assumption. The imputation model included all 40 randomized participants, with group assignment, age, sex, and the pre- and post-intervention data for both the primary and secondary outcomes. Predictive mean matching was used as the imputation method, and five imputed datasets were generated. Final estimates were obtained by pooling the results across the five imputed datasets.

#### Analytical methods

Statistical analysis was performed using IBM SPSS Statistics version 25.0. Continuous variables were tested for normality using the Shapiro–Wilk test. Normally distributed continuous variables were presented as mean ± standard deviation (SD), whereas non-normally distributed continuous variables were reported as median (P25, P75). Categorical variables were described using frequencies and percentages. For between-group comparisons, independent-samples t-tests were used for continuous variables that met the assumptions of normality and homogeneity of variance. When these assumptions were not met, the Mann–Whitney U test was applied. For within-group comparisons before and after the intervention, paired t-tests were used for normally distributed variables, whereas the Wilcoxon signed-rank test was used for non-normally distributed variables. A two-tailed P value of less than 0.05 was considered statistically significant. Both the PP set and the full analysis set based on the ITT principle were used for data analysis. When significant baseline differences were identified in key demographic or clinical variables, such as sex, age, or baseline scores, analysis of covariance (ANCOVA) was performed to adjust the primary outcome. These variables were included as covariates to control for potential confounding effects and to improve the robustness of the intervention effect evaluation.

#### fNIRS data processing and analysis

fNIRS data were processed and analyzed using NirSpark software (version 1.8.8; Danyang Huichuang Medical Equipment Co., Ltd., Danyang, Jiangsu, China). The preprocessing steps were as follows: (1) Raw intensity signals were first converted into optical density signals. Motion artifacts were corrected using a spline interpolation method with the standard deviation threshold set at 6 and the peak threshold set at 0.5 (2). A band-pass filter (0.01–0.2 Hz) was applied to the fNIRS signals to eliminate physiological noise and low-frequency drift that could interfere with the hemodynamic responses (3). The filtered optical density signals were then converted into concentration changes of HbO and HbR using the modified Beer–Lambert law [50, 51]. Compared to HbR, HbO has a higher signal-to-noise ratio, shows stronger correlation with the blood-oxygen-level-dependent signal, and is more sensitive to dynamic changes in cerebral blood flow [50, 52]. Therefore, HbO concentration changes were used as the primary indicator of cortical activation in this study.

Following data preprocessing, block averaging analysis was performed on the three functional tasks using NirSpark software [53–55]. Through the superimposition and averaging of block paradigms, random noise between tasks was minimized, allowing for the extraction of the hemodynamic response function (HRF). The HRF analysis window was defined from − 2 s to 175 s, with the period from − 2 to 0 s used as the baseline and 0 to 35 s corresponding to a single block paradigm. The mean HbO concentration during the task period (0–15 s) was selected as the characteristic value. For each block paradigm, HbO signals were averaged across trials, and the resulting block-averaged HbO responses were used to represent the level of cortical neuronal activation across channels under each task condition. Between-group comparisons of the two groups of children were conducted using independent samples t-tests, while within-group pre- and post-intervention comparisons were analyzed using paired samples t-tests. For the fNIRS analyses, P values from multiple channel or region comparisons were corrected using the false discovery rate (FDR) method.

#### Correlation analysis

Correlation analysis was conducted between brain regions showing statistically significant activation differences and the corresponding changes in motor performance before and after the intervention. Spearman’s rank correlation was used for variables that did not follow a normal distribution, while Pearson’s correlation was applied for normally distributed variables.

## Results

### Participant characteristics, compliance, and attendance

**Fig. 3:**
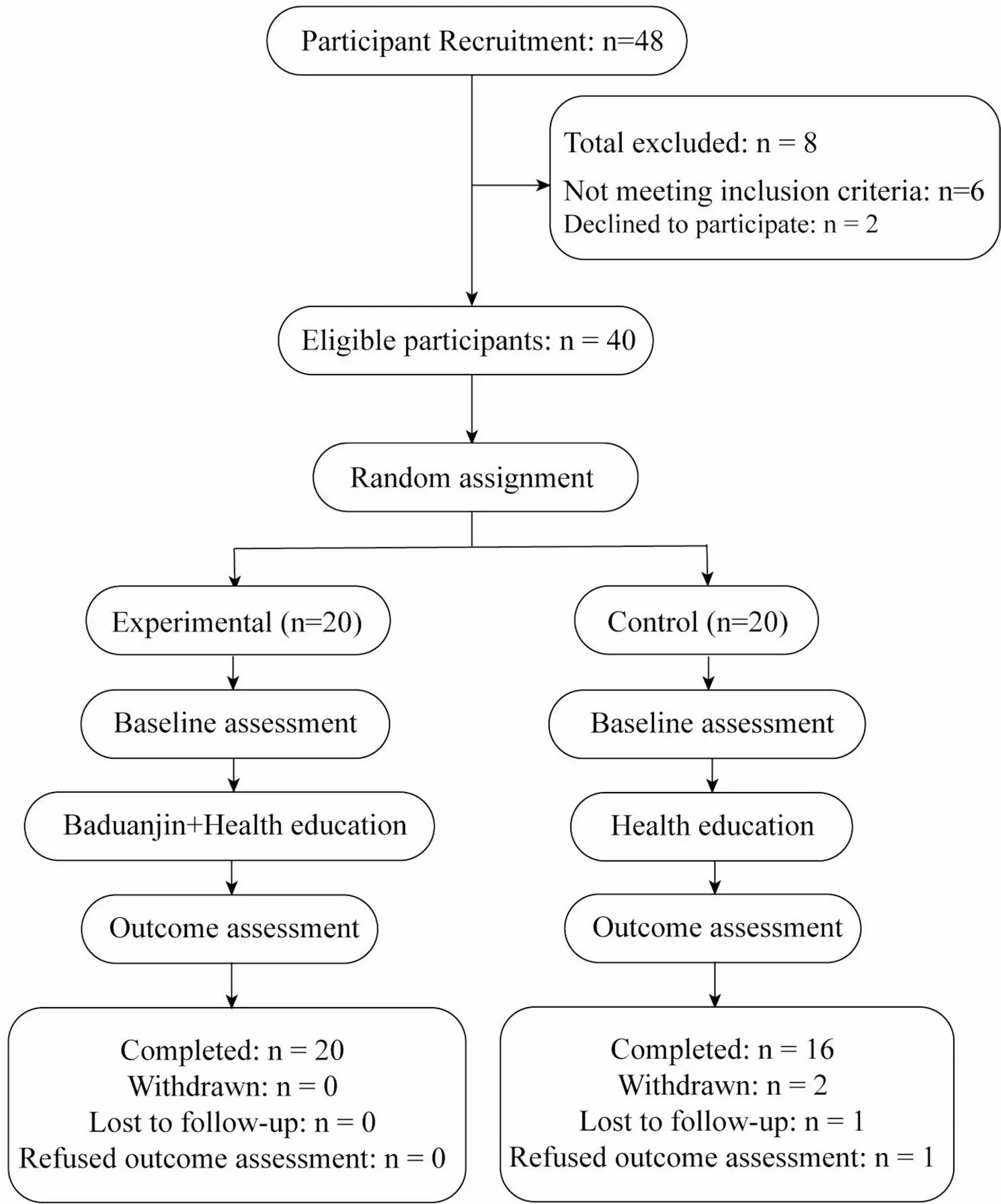
Flowchart of participant enrollment

A total of 48 participants were recruited from the outpatient and inpatient departments of the Department of Pediatrics at Shanghai Fourth People’s Hospital, affiliated with Tongji University. Among them, 8 participants were excluded for not meeting the inclusion criteria, resulting in 40 eligible participants who were randomized equally into the experimental group (*n* = 20) and the control group (*n* = 20). During the study period, four participants in the control group dropped out: two withdrew to pursue other treatments, one was lost to follow-up, and one refused to undergo outcome assessments. All participants in the experimental group completed the full study protocol (see Fig. 3). There were no significant differences between the two groups in terms of age or birth history (*P* ≥ 0.05), but a significant difference was observed in gender distribution (*P* < 0.05) (see Table 2). The number of participants who completed the 8-week intervention was 20 in the experimental group and 16 in the control group, resulting in an overall dropout rate of 10%. Chi-square analysis indicated a statistically significant difference in dropout rates between groups (χ² = 4.444, *P* = 0.035) (see Table 2). There was no significant difference in attendance rates between the experimental group and the control group (Z = − 0.933, *P* = 0.351) (see Table 2).

**Table 2:** Comparison of general characteristics of participants (n [%] / Mean ± SD)

Indicators	Control	Experimental	t/x^2^/Z-value	*P*-value
Age	8.04 ± 1.66	7.79 ± 1.75	-0.472	0.640
Sex				
Male	16(80%)	4(20%)	14.40	< 0.001
Female	4(20%)	16(80%)		
Birth History				
Full-term	10(50%)	13(65%)	0.92	0.337
Preterm	10(50%)	7(35%)		
Compliance				
Completed	20(100%)	16(80%)	4.444	0.035
Dropped Out	0(0%)	4(20%)		
Attendance Rate	77.13 ± 6.30	70.31 ± 22.76	-0.933	0.351

Compliance was defined as completion of the 8-week intervention according to the study protocol and inclusion in the PP analysis set. Attendance rate was calculated as the percentage of completed sessions out of the total number of planned sessions

### Baseline comparison of outcome measures

Before the intervention, no statistically significant differences were observed between the two groups in the MABC-2 total score or in the three subdomains: manual dexterity, aiming and catching, and balance (*P* ≥ 0.05). Similarly, static balance indicators—sway path length and the sway area—did not differ significantly between the groups (*P* ≥ 0.05). In terms of dynamic postural control, there was no significant difference in the stability limit index (*P* ≥ 0.05). These results indicate that the two groups were comparable across all outcome measures at baseline (see Table 3).

**Table 3:** Baseline comparison of outcome measures between two groups (Mean ± SD / Median [P25, P75])

Indicators	Experimental(*n* = 20)	Control(*n* = 20)	t/Z-value	*P*-value
MABC-2^*^	56.00(51.25,66.00)	58.00(45.75,63.75)	-0.678	0.512
manual dexterity	19.10 ± 5.57	16.80 ± 6.45	1.206	0.235
aiming and catching ^*^	11.00(9.00,12.00)	12.00(11.00,13.75)	-1.656	0.102
balance ^*^	28.50(25.00,30.75)	27.5(17.50,30.75)	-0.380	0.718
Static balance				
Sway area ^*^	150.00(103.00,278.75)	225.00(173.00,292.25)	-1.678	0.096
Sway path length	431.80 ± 142.05	416.70 ± 94.70	0.396	0.695
Dynamic postural stability				
stability limit index	65.62 ± 15.25	57.27 ± 15.33	1.727	0.092

*Data are not normally distributed; the Mann-Whitney U test for two independent samples was used

### Comparison of post-intervention outcome measures between groups

#### Comparison of MABC-2 scores after intervention

Post-intervention analysis based on the PP dataset showed that the total MABC-2 score was significantly higher in the experimental group compared to the control group (Z = − 3.017, *P* = 0.002). Among the subdomains, the experimental group scored significantly higher in aiming and catching (t = 2.457, *P* = 0.019) and balance (Z = − 2.614, *P* = 0.008), while no significant difference was found in manual dexterity (t = 1.811, *P* = 0.079). Analysis using the ITT dataset revealed consistent results. After the intervention, the experimental group demonstrated significantly higher total MABC-2 scores (Z = − 3.266, *P* = 0.001), as well as significantly higher scores in aiming and catching (t = 2.976, *P* = 0.005) and balance (Z = − 2.870, *P* = 0.004), compared to the control group. Manual dexterity scores remained non-significant between groups (t = 1.954, *P* > 0.05) (see Table 4). The results showed that, compared with the control group, Baduanjin intervention significantly improved the MABC-2 scores of children with DCD in the experimental group, as well as their performance in the subdomains of aiming and catching and balance.

**Table 4:** Comparison of post-intervention MABC-2 scores between two groups of children (Mean ± SD / Median [P25–P75])

	Indicators	Experimental	Control	t/*Z*-value	*P*-value
PP (E: *n* = 20/ C: *n* = 16)	MABC-2^*^	70.50(66.25,75.75)	60.00(40.00,69.50)	-3.017	0.002
	manual dexterity	21.10 ± 5.46	17.56 ± 6.26	1.811	0.079
	aiming and catching	15.75 ± 2.94	13.44 ± 2.63	2.457	0.019
	balance ^*^	34.50(30.25,38.00)	26.00(14.50,33.00)	-2.614	0.008
ITT (E: *n* = 20/ C: *n* = 20)	MABC-2 ^*^	70.50(66.25,75.75)	59.10(43.25,67.75)	-3.266	0.001
	manual dexterity	21.10 ± 5.46	17.69 ± 5.60	1.954	0.058
	aiming and catching	15.75 ± 2.94	13.22 ± 2.43	2.976	0.005
	balance ^*^	34.50(30.25,38.00)	26.55(17.23,32.25)	-2.870	0.004

*Data are not normally distributed; the Mann-Whitney U test for two independent samples was used; E= Experimental; C= Control

Given the baseline difference in gender distribution between groups, ANCOVA was conducted to adjust for potential confounding effects of gender and baseline scores on post-intervention outcomes. In this model, the post-intervention total MABC-2 score was set as the dependent variable, group as the fixed factor, and both gender and baseline MABC-2 score (MABC2_pre) as covariates. Interaction terms group*gender and group*MABC2_pre were included to test for slope homogeneity and moderating effects. ANCOVA results (see Table S2) indicated that, after controlling for baseline scores and gender, the difference in post-intervention MABC-2 total scores between the experimental and control groups remained statistically significant (F = 4.517, *P* = 0.042), suggesting an independent effect of the intervention on motor function improvement. The main effect of gender was not significant (F = 1.406, *P* = 0.245), indicating that gender had a limited influence on post-intervention outcomes. The interaction terms group*MABC2_pre (F = 0.578, *P* = 0.453) and group*gender (F = 1.746, *P* = 0.196) were also not significant, supporting the assumption of homogeneity of regression slopes and indicating consistent effects of the covariates across groups. The model demonstrated a good fit, with an adjusted R² of 0.893. After adjusting for covariates, the estimated marginal mean of the MABC-2 score in the experimental group was 66.82 (SE = 1.20), compared to 57.06 (SE = 1.38) in the control group, yielding a mean difference of 9.76 points (95% CI: 6.02–13.50, *P* < 0.001). These findings indicate a significant intervention effect that is not substantially influenced by gender imbalance.

#### Comparison of ProKin 254 balance test after intervention

Post-intervention balance assessment data from the Pro-Kin 254 system did not follow a normal distribution; therefore, non-parametric tests were used for statistical analysis. Both PP and ITT analyses demonstrated that the experimental group showed statistically significant improvements in both key indicators of static balance compared to the control group. Specifically, the experimental group exhibited significantly reduced sway area (PP: Z = − 3.535, *P* < 0.001; ITT: Z = − 3.856, *P* < 0.001) and sway path length (PP: Z = − 3.757, *P* < 0.001; ITT: Z = − 4.139, *P* < 0.001). Regarding dynamic postural control, the experimental group also achieved significantly higher stability limit index compared to the control group (PP: Z = − 2.833, *P* = 0.004; ITT: Z = − 2.786, *P* = 0.004). These findings indicated that, compared to the control group, the experimental group experienced notable improvements in both static balance and dynamic postural control following the 8-week training program (see Table 5).

**Table 5:** Comparison of post-intervention pro-kin254 balance test between two groups (Median [P25–P75])

	Indicators	Experimental	Control	*Z*-value	*P*-value
PP (E: *n* = 20/ C: *n* = 16)	Static balance				
	Sway area ^*^	114.50(84.00,135.00)	249.50(182.00,329.00)	-3.535	< 0.001
	Sway path length ^*^	267.50(214.00,292.75)	434.00(334.25,513.50)	-3.757	< 0.001
	Dynamic postural stability				
	stability limit index ^*^	72.30(65.53,79.83)	60.65(51.28,69.93)	-2.833	0.004
ITT (E: *n* = 20/ C: *n* = 20)	Static balance				
	Sway area ^*^	114.50(84.00,135.00)	238.50(190.25,314.00)	-3.856	< 0.001
	Sway path length ^*^	267.50(214.00,292.75)	419.00(340.00,490.00)	-4.139	< 0.001
	Dynamic postural stability				
	stability limit index ^*^	72.30(65.53,79.83)	61.65(53.50,71.23)	-2.786	0.004

*Data are not normally distributed; the Mann-Whitney U test for two independent samples was used

### Comparison of pre- and post-intervention outcome measures within groups

In the experimental group, both PP and ITT analyses revealed significant improvements following the intervention. Specifically, post-intervention scores showed significant increases in the MABC-2 total score (PP/ITT: t = 11.619, *P* < 0.001), aiming and catching (PP/ITT: t = 5.396, *P* < 0.001), and balance (PP/ITT: t = 6.781, *P* < 0.001). Additionally, significant improvements were observed in Pro-Kin 254 balance indicators, including sway area (t = − 2.128, *P* = 0.033), sway path length (t = − 4.791, *P* < 0.001), and stability limit index (t = 3.322, *P* = 0.004). However, no statistically significant change was found in manual dexterity scores (*P* ≥ 0.05) (see Table S3). In contrast, within-group comparisons for the control group showed no significant improvements in the MABC-2 total score, manual dexterity, aiming and catching, or balance, nor in sway area or sway path length (*P* ≥ 0.05), based on both PP and ITT analyses. However, ITT analysis revealed a statistically significant increase in the stability limit index after the intervention (t = 2.378, *P* = 0.028) (see Table S3). The results showed that, compared to pre-intervention, children in the experimental group exhibited significant improvements in MABC-2 scores, static balance, and dynamic postural control after the intervention. Although children in the control group demonstrated a statistically significant improvement in dynamic postural control in the ITT dataset, no significant improvements were observed across any outcome measures in the PP dataset.

### fNIRS results

#### Comparison of post-intervention cortical activation between groups

**Fig. 4:**
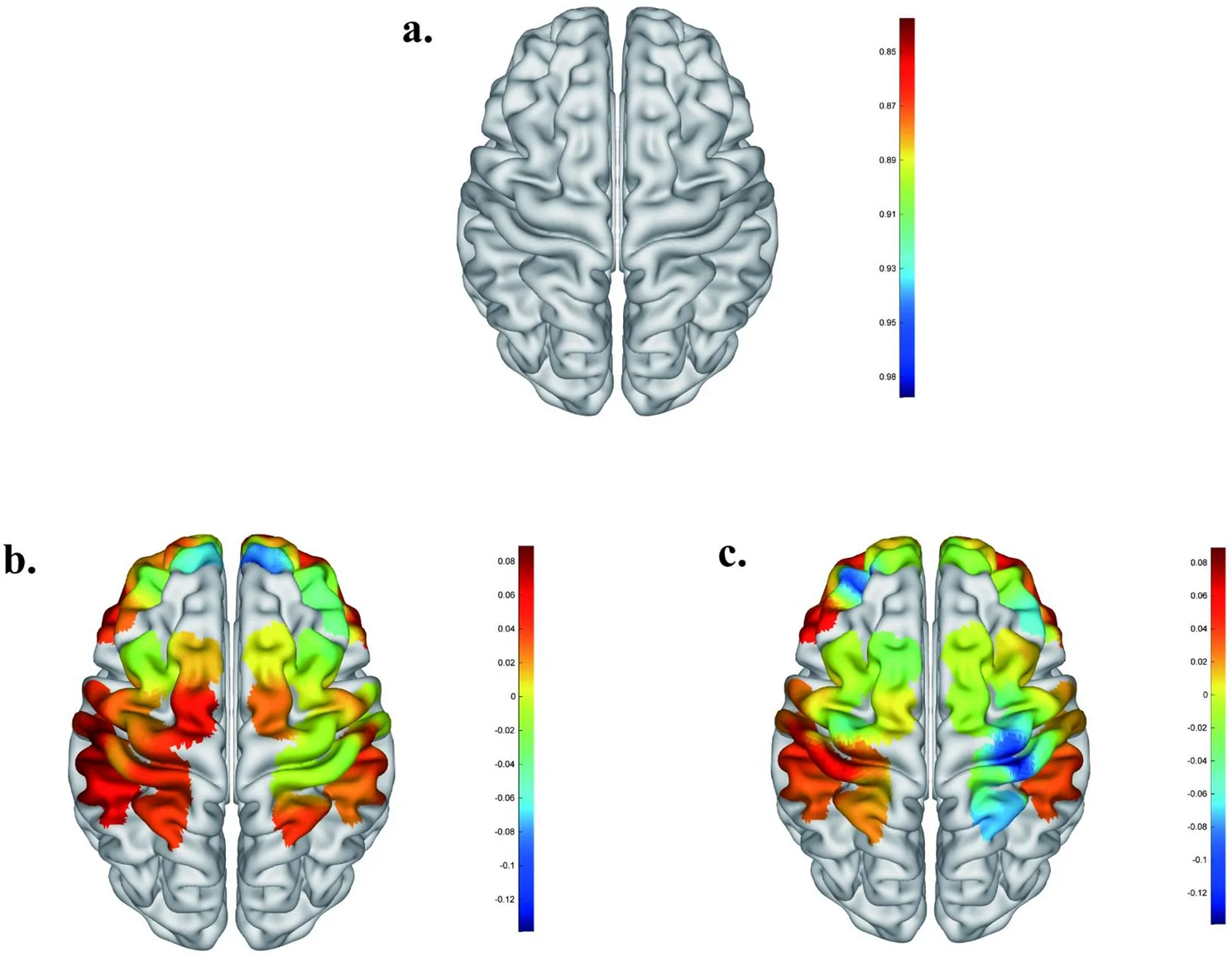
Brain activation maps during the drawing trail task after intervention in two groups of children. (Note: (**a**) Significant brain regions showing differences in activation between the two groups; (**b**) Brain activation map of the experimental group; (**c**) Brain activation map of the control group.)

**Fig. 5:**
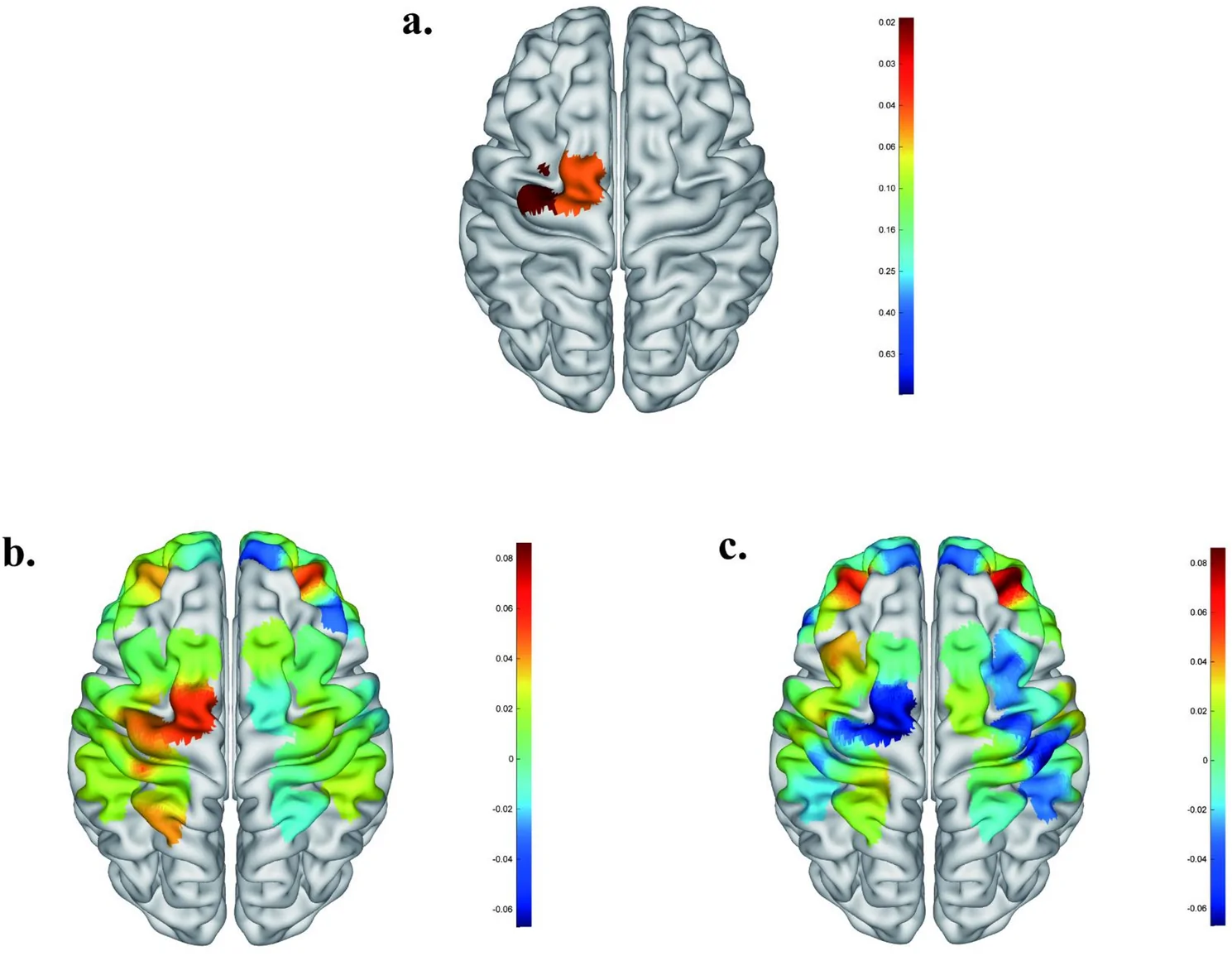
Brain activation maps during the aiming and catching task after intervention in two groups of children. (Note: (**a**) Significant brain regions showing differences in activation between the two groups; (**b**) Brain activation map of the experimental group; (**c**) Brain activation map of the control group.)

**Fig. 6:**
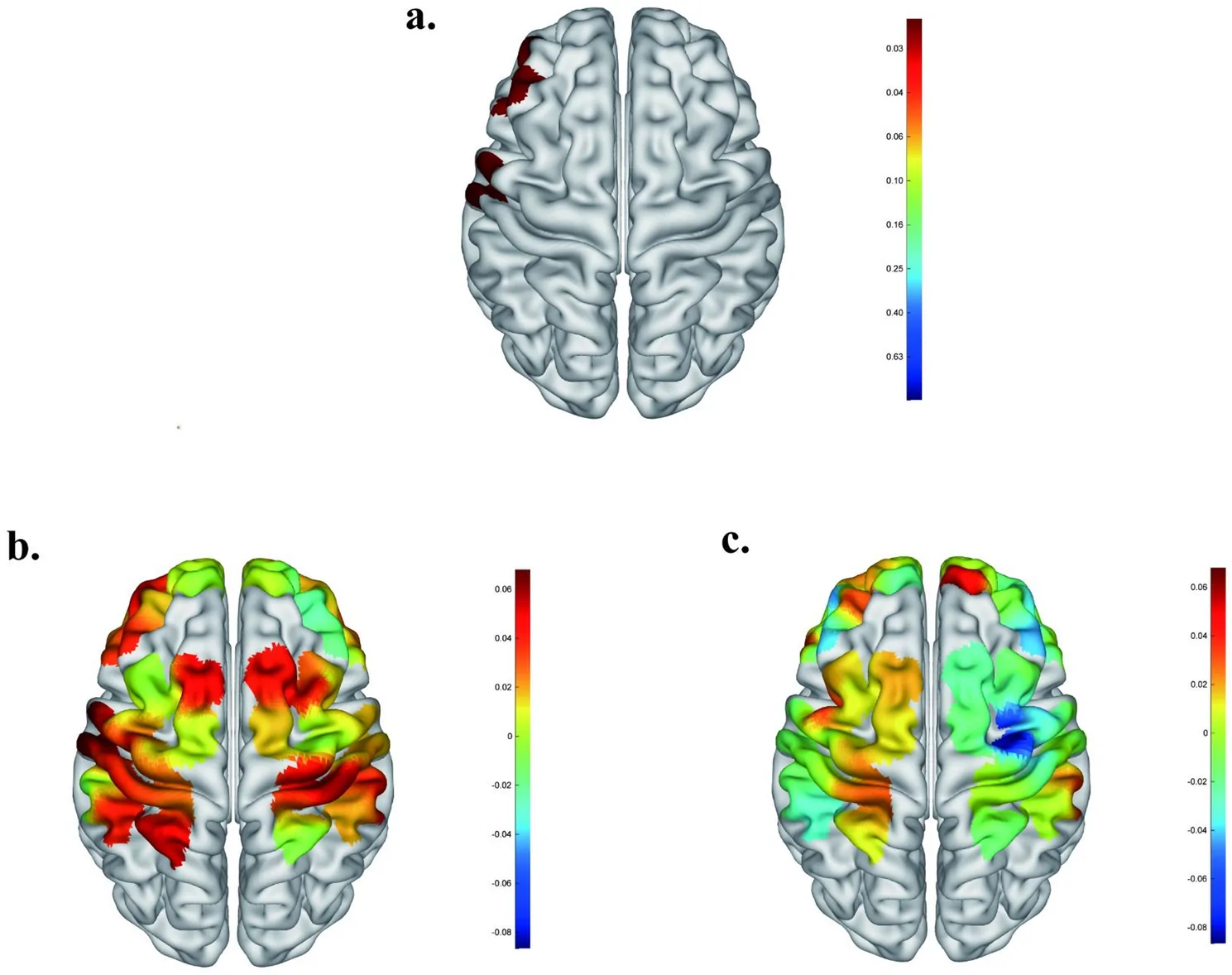
Brain activation maps during the one-leg balance task after intervention in two groups of children. (Note: (**a**) Significant brain regions showing differences in activation between the two groups; (**b**) Brain activation map of the experimental group; (**c**) Brain activation map of the control group.)

Post-intervention fNIRS analysis showed no statistically significant differences in cortical activation between the experimental and control groups during the drawing trail task across the M1, PMC + SMA, S1, S2, and PFC regions (FDR-adjusted *P* ≥ 0.05) (Fig. 4a). The corresponding activation maps for the experimental and control groups during this task are shown in Fig. 4b and c, respectively. In the aiming and catching task, the experimental group exhibited significantly greater activation in the left PMC + SMA compared to the control group following the intervention (CH31: t = 4.019, FDR-adjusted *P* = 0.015; CH32: t = 3.414, FDR-adjusted *P* = 0.041) (Fig. 5a). Activation maps for the experimental and control groups during this task are displayed in Fig. 5b and c. During the one-leg balance task, children in the experimental group showed significantly greater activation in several regions of the left hemisphere compared to the control group, including the frontopolar cortex (FPC) and inferior frontal gyrus (IFG) (t = 3.703, FDR-adjusted *P* = 0.018), DLPFC (t = 3.546, FDR-adjusted *P* = 0.019), and M1 and PMC + SMA (t = 3.912, FDR-adjusted *P* = 0.018) (Fig. 6a). The corresponding brain activation maps for the experimental and control groups during this task are presented in Fig. 6b and c, respectively. The results indicated that, compared to the control group, Baduanjin intervention significantly enhanced activation in motor-related brain regions in the experimental group during the execution of the aiming and catching and one-leg balance tasks.

#### Comparison of pre- and post-intervention cortical activation within groups

In the experimental group, fNIRS analysis revealed no significant changes in cortical activation across motor-related regions during the drawing trail task before and after the intervention (FDR-adjusted *P* ≥ 0.05) (see Figure S3a). During the aiming and catching task, the experimental group showed significantly increased activation in the left PMC + SMA following the intervention (t = 4.014, FDR-adjusted *P* = 0.037) (see Figure S4a). In the one-leg balance task, post-intervention results demonstrated significantly enhanced activation in multiple brain regions: FPC (t = 3.519, FDR-adjusted *P* = 0.028), left DLPFC and IFG (t = 4.956, FDR-adjusted *P* = 0.002), left M1 and PMC + SMA (t = 5.226, FDR-adjusted *P* = 0.002), and left S1 and S2 (t = 4.314, FDR-adjusted *P* = 0.006) (see Figure S5a). In contrast, within-group analysis of the control group showed no significant pre-to-post changes in cortical activation across motor-related regions during any of the three functional tasks (FDR-adjusted *P* ≥ 0.05) (see Figures S6–S8). These results indicate that, in the pre- and post-intervention comparison, Baduanjin training significantly enhanced activation in motor-related brain regions in the experimental group during the execution of the aiming and catching and one-leg balance tasks, whereas no such improvements were observed in the control group across any of the functional tasks.

### Correlation between motor performance and cortical activation

**Fig. 7:**
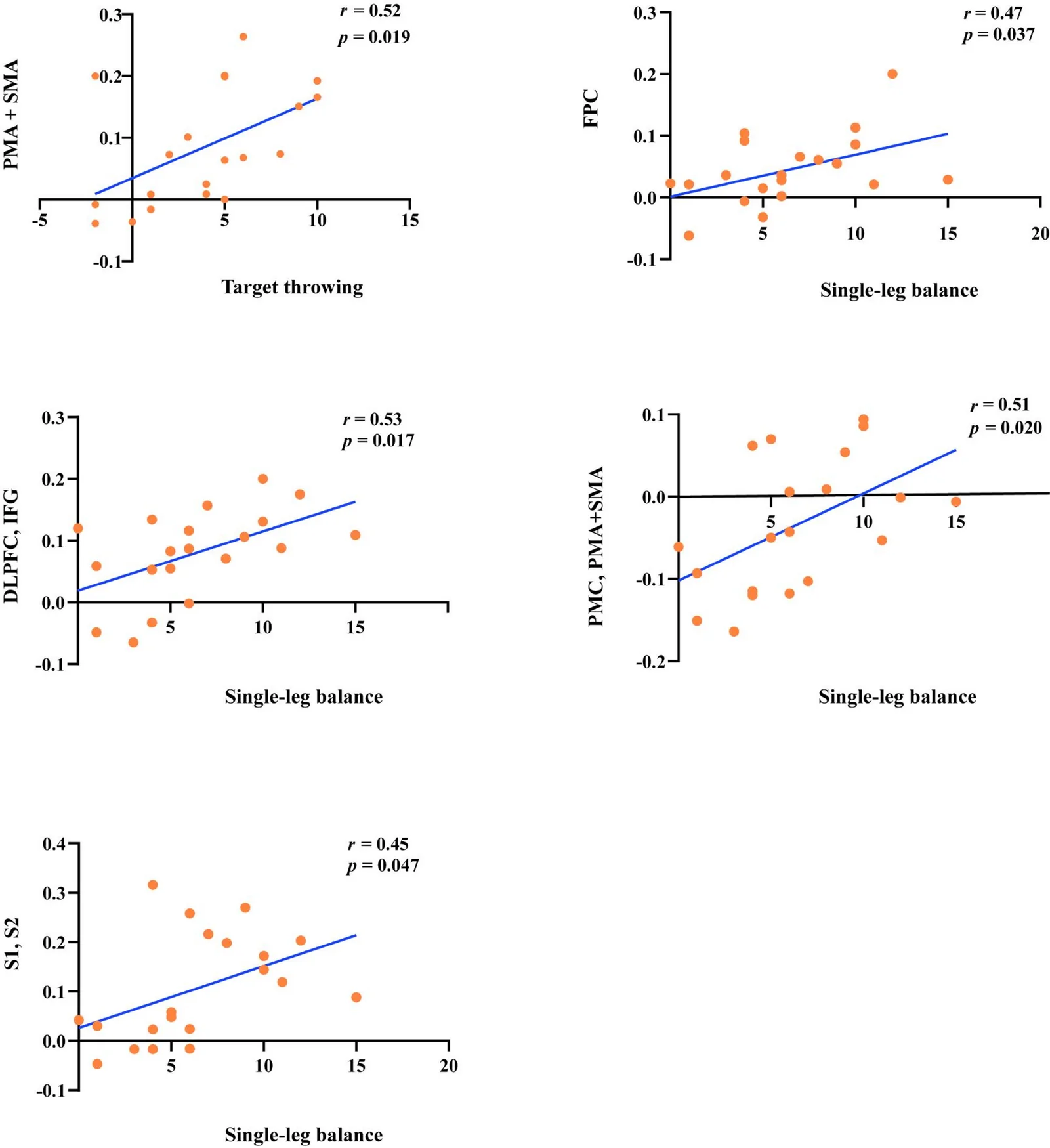
Correlation analysis between motor performance and brain activation

Correlation analysis was conducted between brain regions that showed significant activation changes and motor performance metrics that demonstrated significant improvement after the intervention. The results indicated a significant positive correlation between performance in the aiming and catching task and activation in the PMC + SMA region (*r* = 0.52, *P* = 0.019). For the single-leg balance task, performance improvements were significantly positively correlated with activation in the FPC (*r* = 0.47, *P* = 0.037), left DLPFC (*r* = 0.53, *P* = 0.017), IFG (*r* = 0.53, *P* = 0.017), M1 (*r* = 0.51, *P* = 0.020), PMC + SMA (*r* = 0.51, *P* = 0.020), and the S1 and S2 (*r* = 0.45, *P* = 0.047) (See Fig. 7). The results suggest that the improvement in motor function among children with DCD following Baduanjin training is associated with enhanced activation in motor-related brain regions.

## Discussion

This study represents the first clinical investigation into the effects of the traditional Chinese exercise Baduanjin on motor function in children with DCD. The findings suggest that Baduanjin training has a significantly positive impact on motor performance in children with DCD. Among the primary outcome measures, Baduanjin training was associated with significantly improved MABC-2 total scores. Notably, significant improvements were observed in the subdomains of aiming and catching and balance, whereas the improvement in manual dexterity did not reach statistical significance. In terms of training intensity and dosage, the present study employed an 8-week intervention with five sessions per week, yielding a total of 40 sessions and thus a relatively adequate training dose. This intervention duration was within the range reported in previous Baduanjin studies [17–19, 22, 56] that showed improvements in balance and motor-related function, suggesting that the present findings may provide useful reference. Nevertheless, such comparisons should be interpreted cautiously, given the heterogeneity across studies in participant characteristics and outcome measures. The enhancement in aiming and catching may be related to the characteristics of Baduanjin. This form of exercise emphasizes full-body coordinated movements with smooth, rhythmic transitions, requiring the integration of multiple sensory modalities such as visual, vestibular, and proprioceptive input. This multisensory integration promotes effective coordination between sensory perception and motor output. Previous studies have shown that such integration plays a critical role in improving the precision and reliability of motor control and learning processes [57–59]. Therefore, long-term practice of Baduanjin may enhance sensorimotor coordination in complex tasks among children with DCD, particularly by improving visual–vestibular–proprioceptive integration and facilitating the alignment between sensory feedback and motor execution. This mechanism may partly contribute to the observed improvements in aiming and catching. Similarly, the observed improvements in balance may be partly related to key elements inherent in Baduanjin training. The practice includes frequent weight-shifting from multiple angles, single-leg stances, and coordinated upper and lower limb movements. These movement patterns may help stimulate and strengthen postural control mechanisms, thereby supporting balance performance under various motor demands in children with DCD. However, the improvement in manual dexterity did not reach statistical significance following the Baduanjin intervention. This may be attributed to the nature of Baduanjin, which primarily involves coordinated movements of large muscle groups and the core musculature, with limited emphasis on high-frequency, finely regulated small muscle control activities [60]. As a result, its direct effect on enhancing fine motor performance in the upper limbs may be relatively limited. In addition, fine motor skills depend on more complex sensorimotor integration [61–65], and both their development and the associated neural adaptations may require longer-term training [66]. Previous research suggests that inducing neuroplastic changes—particularly in tasks involving small muscle coordination and high-level sensorimotor integration—often necessitates high-frequency, sustained training over several months to produce stable structural and functional cortical reorganization [67]. In this study, the 8-week intervention may have been sufficient to elicit plastic changes in the gross motor control system, but likely insufficient to induce significant reorganization in cortical regions associated with fine motor control, such as the hand area of M1, DLPFC, and parietal–motor association regions. Future studies may consider integrating more targeted fine motor training components alongside Baduanjin practice, such as hand-grip exercises, fine motor task training, and enhanced tactile–motor feedback strategies. These approaches may promote greater activation and reorganization of motor-related cortical networks involved in hand function, thereby enabling more comprehensive improvement in motor performance among children with DCD.

In the Pro-kin balance test, children with DCD demonstrated significant improvements in both static balance and dynamic postural control following Baduanjin training. During the Baduanjin practice, the horse stance—which requires sustained knee flexion—places the lower limbs in a continuous weight-bearing state. From a biomechanical perspective, this posture may increase the engagement of major lower limb muscle groups, including the quadriceps femoris, hamstrings, and triceps surae. These muscles undergo regular concentric and eccentric contractions throughout the repetitive movement patterns, and the mechanical stress coupled with metabolic adaptations induced by such activity may contribute to neuromuscular adaptation and improved lower-limb strength or endurance. Additionally, the diverse body movements involved in Baduanjin promote continuous shifting of the body’s center of gravity in multiple directions—anterior-posterior, medial-lateral, and vertical. These dynamic shifts may help improve overall coordination and postural stability. The improvement in lower limb core muscle strength and postural control brought about by Baduanjin training may help children with DCD maintain a stable upright posture more effectively during static conditions. Beyond the neuromuscular biomechanical mechanisms, improvements in Dynamic postural control may also involve more complex pathways, including multisensory feedback—such as visual, vestibular, and proprioceptive inputs—as well as higher-order cognitive processes like attention and executive function [68–70]. In Baduanjin practice, the movement “Bouncing on the toes” may stimulate lower limb proprioceptors through the body’s own gravitational load, thereby supporting proprioceptive input and neuromuscular control. This may facilitate more effective postural adjustments in response to environmental changes during movement. Children with DCD often exhibit vestibular dysfunction, which impairs their ability to maintain balance during dynamic activities [70, 71]. The movement “Wise owl gazes backwards” incorporates rotational motions of the head and neck, which may stimulate the vestibular system and promote its adaptive regulation, thereby supporting the integration of proprioceptive, vestibular, and visual information. Furthermore, numerous studies have demonstrated that regular, long-term Baduanjin practice significantly improves cognitive functions, including attention and executive control [72–74]. Therefore, by integrating multisensory feedback with higher-level cognitive functions, Baduanjin may play a beneficial role in enhancing dynamic postural control in children with DCD.

fNIRS-based brain function assessments revealed that, following Baduanjin training, children with DCD exhibited significantly enhanced activation in multiple motor-related cortical regions during both target-throwing and single-leg balance tasks. In the target-throwing task, Baduanjin training significantly increased activation in the PMC + SMA, and this heightened activation was positively correlated with improved performance scores in the task. The PMC + SMA are key regions involved in motor planning and execution [30, 75, 76]. Specifically, the PMC is responsible for integrating sensory feedback and planning motor actions; it synthesizes information from multiple sensory channels to generate appropriate motor commands [77], and plays a critical role in motor sequence learning [78]. The SMA is essential for organizing complex movement sequences and for the voluntary control of movement [79]. Therefore, the increased activation observed in these regions may indicate training-related functional changes in neural processes relevant to goal-directed motor control. However, increased cortical activation should not be automatically interpreted as beneficial neuroplasticity, as it may also reflect compensatory recruitment or greater task demands during task performance. Because the present study did not directly assess structural or synaptic neuroplasticity, these findings should be considered indirect evidence only. In the one-leg balance task, children with DCD who underwent Baduanjin training exhibited significantly increased activation in the left FPC, DLPFC, IFG, M1, and PMC + SMA, compared to the control group. Moreover, the level of activation in these regions was positively correlated with improvements in single-leg balance ability. The DLPFC, FPC, and IFG are all components of the PFC, which is involved in higher-order cognitive control, motor planning, and attentional regulation [80]. Deficits in PFC function in children with DCD have been associated with difficulties in tasks requiring cognitive-motor integration [81, 82]. The increased PFC activation observed post-training may reflect altered recruitment of cognitive control resources during balance-related tasks. At the same time, this pattern could also represent compensatory activation or increased cognitive-motor demands, rather than a straightforwardly beneficial neural adaptation. Additionally, Baduanjin training was also associated with enhanced activation in the M1 and M2 region, which may have contributed to improved balance performance. Although no significant between-group differences were observed in the activation of the S1 and S2, within-group comparisons in the experimental group revealed significant post-training increases in S1 and S2 activation, which were also positively correlated with improvements in balance. The S1 and S2 regions play a central role in processing and integrating somatosensory input from various parts of the body through complex neural networks, thereby enhancing body posture awareness and motor control [83]. Strengthened integration in these regions may contribute not only to better balance and coordination but also to improved postural stability in children with DCD. The enhanced co-activation of M1, M2, S1, and S2 may reflect changes in sensorimotor information processing and integration during postural regulation following Baduanjin training [83]. Nevertheless, these interpretations remain tentative, and future studies using more direct physiological and neurobiological measures are needed to determine whether the observed activation increases primarily reflect beneficial adaptation, compensatory recruitment, or greater task-related demands.

Although the present study demonstrates that Baduanjin training can effectively improve motor coordination and balance in children with DCD and enhances activation in relevant brain regions at the neural level, several limitations should be acknowledged. First, four participants in the control group were lost to follow-up during the outcome assessment phase, which may introduce some bias to the primary outcomes. To address this, we conducted sensitivity analyses using extreme-value imputation to simulate the missing data. The results showed that even under the most favorable assumption for the control group, the post-intervention mean score reached only 58.45, still significantly lower than the experimental group’s mean of 69.15. These findings suggest that the missing data were unlikely to overturn the primary outcome, although some attrition-related bias cannot be fully excluded. While the sensitivity analysis supported the robustness of the primary outcome, differential attrition may still have affected the stability of the secondary outcomes, and these findings should therefore be interpreted with caution. Second, Although baseline sex distribution differed between groups, the adjusted analysis did not show a significant main effect of sex or a significant group-by-sex interaction for the MABC-2 outcome. However, because sex was not uniformly adjusted for across the other outcome analyses, residual confounding cannot be completely excluded. Third, all participants were recruited from a single geographic region, resulting in a relatively homogeneous sample and limiting the generalizability of our findings to children from different cultural, socioeconomic, or clinical backgrounds. In addition, no long-term follow-up was conducted, so it remains unclear whether the observed improvements in motor and cortical function can be sustained after the intervention. Therefore, our findings should be considered preliminary rather than broadly generalizable. Future multi-center studies with more diverse samples, more comprehensive sociodemographic data, and longer follow-up are needed to further validate the broader applicability and durability of Baduanjin training. Fourth, fine motor skills involve more complex neuromuscular control mechanisms, and the improvements achieved through Baduanjin training alone may be limited in this domain. Future studies could explore integrated intervention approaches that combine Baduanjin with occupational therapy or fine motor training programs to more effectively enhance fine motor abilities in children with DCD. Lastly, although the randomized controlled trial design helped reduce baseline differences between groups, individual characteristics such as DCD severity, comorbidities, cognitive functioning, family support, and physical development or maturation may still have influenced training outcomes. The present study did not include a detailed analysis of how these factors might moderate the effects of the intervention. In addition, although home-based practice was monitored through parental supervision, video submission, and training logs, the actual quality and consistency of home practice could not be fully controlled. Future studies should adopt more individualized analytical strategies to examine differential responses to Baduanjin training among children with different profiles, thereby informing more personalized and targeted intervention approaches.

From a clinical perspective, the present findings suggest that Baduanjin may be a feasible adjunct to rehabilitation programs for children with DCD. As a structured and relatively low-cost exercise approach, it may be particularly useful for improving motor coordination and balance in both clinical and home-based settings. The inclusion of parent-supervised home practice may also help extend training beyond the clinic and support rehabilitation continuity. However, given the limited improvement in manual dexterity observed in this study, Baduanjin should be considered a complementary intervention rather than a stand-alone solution, and may need to be combined with more targeted fine motor training to address the broader rehabilitation needs of children with DCD.

## Conclusion

This study evaluated the effects of Baduanjin training on motor function in children with DCD from both behavioral and neurophysiological perspectives. The results showed that, compared with the control group, Baduanjin training significantly improved overall motor coordination and balance, including the MABC-2 total score, aiming and catching, balance, and both static and dynamic postural control. In contrast, the improvement in manual dexterity did not reach statistical significance, suggesting that its effect on fine motor function may be limited. Future studies may therefore consider combining Baduanjin with more targeted fine motor training or occupational therapy. The fNIRS findings showed greater activation in motor-related cortical regions during the target-throwing and one-leg balance tasks after Baduanjin training, and some of these activation changes were associated with improvements in motor performance. These findings provide preliminary neurophysiological support for the functional benefits of Baduanjin.

Overall, Baduanjin may serve as a useful adjunct intervention in rehabilitation programs for children with DCD, particularly for improving gross motor coordination and balance. However, the findings should be interpreted in light of the relatively short intervention duration, the small single-center sample, and the lack of long-term follow-up. Future studies should adopt multi-center designs, include longer follow-up, and explore combined interventions to further evaluate its long-term effects and broader applicability.

## Supplementary Information

Supplementary Material 1.

Supplementary Material 2.

Supplementary Material 3.

Supplementary Material 4.

Supplementary Material 5.

Supplementary Material 6.

Supplementary Material 7.

## Data Availability

The data that support the findings of this study are available from the corresponding author upon reasonable request.

## Abbreviations

DCD: Developmental coordination disorder
DLPFC: Dorsolateral prefrontal cortex
PMC/M1: Primary motor cortex
PMC + SMA/M2: Premotor cortex and Supplementary motor area
S1: Primary somatosensory cortex
S2: Secondary somatosensory cortex
PFC: Prefrontal cortex
MRI: Magnetic resonance imaging
EEG: Electroencephalography
fNIRS: Functional near infrared spectroscopy
HbO: Oxygenated hemoglobin
HbR: Hemoglobin
DSM-V: Diagnostic and statistical manual of mental disorders, fifth edition
MABC-2: Motor assessment battery for children - second edition
PP: Per protocol
ITT: Intention-to-treat
ROI: Region of interest
SD: Standard deviation
ANCOVA: Analysis of covariance
HRF: Hemodynamic response function
FPC: Frontopolar cortex
IFG: Inferior frontal gyrus
