# Supplementary material for: Baduanjin improves motor function and cortical activation in children with developmental coordination disorder: a randomized controlled trial
Source: BMC Complement Med Ther. 2026 Aug 13;26:343. doi: 10.1186/s12906-026-05509-w (PMC13749755; doi:10.1186/s12906-026-05509-w)
Supplement: Supplementary file 2 [file 12906_2026_5509_MOESM2_ESM.docx]

**Supporting information 2.**

**Table S1. Detailed description of the standardized Baduanjin exercise protocol.**

| **Segment** | **Movement Name** | **Detailed Description** |
| --- | --- | --- |
| Preparation | Regulating Breath and Focusing the Mind | Participants stood naturally with feet shoulder-width apart, knees slightly flexed, arms relaxed at the sides, and gaze directed forward. Breathing was adjusted to a calm, steady rhythm to promote physical and mental relaxation. |
| Movement 1 | Holding up the Sky to Regulate the Triple Burner | From the abdomen, both hands were lifted upwards with palms facing up, fully extending the arms overhead. The head tilted slightly backward with eyes following the hands. After a brief pause, arms were lowered outward to the sides to the starting position. Repeated 6 times. |
| Movement 2 | Drawing the Bow Left and Right as if Shooting a Vulture | In a horse stance, hands crossed in front of the chest (left hand outside). The left hand pushed horizontally outward with an open palm while the right hand pulled backward in a hook gesture, simulating archery. The head turned left, eyes fixed on the left fingertips. The movement was alternated to the right side, 3 repetitions per side. |
| Movement 3 | Separating Heaven and Earth to Regulate the Spleen and Stomach | Alternating sides, one hand was raised above the head with the palm facing up, while the other hand pressed down beside the hip, generating a vertical traction force. Alternated 6 times per side. |
| Movement 4 | Looking Backwards to Eliminate Five Fatigues and Seven Injuries | With feet stationary, the head and torso rotated to the left rear, eyes gazing behind, while both hands pressed downward beside the hips. The movement was alternated to the right side, repeated 3 times per side. |
| Movement 5 | Swaying the Head and Swinging the Tail to Reduce Heart Fire | In a horse stance, hands rested on the thighs, torso leaned forward. The head swayed to the left rear while hips moved to the right front in a circular arc. Reversed direction for the opposite side. Performed 3 times per side. |
| Movement 6 | Touching the Toes to Strengthen the Kidneys and Waist | Both hands raised overhead with palms facing forward, torso bent forward to touch toes or ground, paused briefly, then hands slid up along the back of legs to support lower back during return to standing. Repeated 6 times. |
| Movement 7 | Clenching the Fists and Glaring to Enhance Strength | In horse stance, fists clenched at the waist (palms up). One fist punched forward with rotation so palm faced downward, accompanied by wide-open eyes. Fist was retracted and alternated to the other hand. 3 repetitions per side. |
| Movement 8 | Bouncing on the Toes to Cure All Illnesses | Standing with feet together, arms relaxed, heels were raised slowly with weight on forefoot, held briefly, and then dropped lightly to induce vibration through the body. Repeated 7 times. |
| Closing | Guiding Qi Back to the Origin | Both hands crossed and placed in front of the lower abdomen. Participants stood quietly, focused on steady breathing to conclude the session. |

### **Figure S2. **Children’s Baduanjin scoring sheet.****
