# Supplementary material for: Baduanjin improves motor function and cortical activation in children with developmental coordination disorder: a randomized controlled trial
Source: BMC Complement Med Ther. 2026 Aug 13;26:343. doi: 10.1186/s12906-026-05509-w (PMC13749755; doi:10.1186/s12906-026-05509-w)
Supplement: Supplementary file 3 [file 12906_2026_5509_MOESM3_ESM.docx]

**Supporting information 3.**

**Table S2. Comparison of post-intervention MABC-2 scores between groups controlling for gender and baseline (ANCOVA).**

| **Variable** | **Mean ± SE** | **F** | **p-value** | **95% CI** |
| --- | --- | --- | --- | --- |
| Intervention group | 66.82 ± 1.20 |  |  | 64.37 – 69.27 |
| Control group | 57.06 ± 1.38 |  |  | 54.24 – 59.89 |
| Group (main effect) |  | 4.517 | 0.042 |  |
| Gender (covariate) |  | 1.406 | 0.245 |  |
| MABC2_pre (covariate) |  | 175.509 | <0.001 |  |
| Group × Gender |  | 1.746 | 0.196 |  |
| Group × MABC2_pre |  | 0.578 | 0.453 |  |

Note: Estimated marginal means based on MABC2_pre = 55.53, gender = 1.44.
