# Supplementary material for: Baduanjin improves motor function and cortical activation in children with developmental coordination disorder: a randomized controlled trial
Source: BMC Complement Med Ther. 2026 Aug 13;26:343. doi: 10.1186/s12906-026-05509-w (PMC13749755; doi:10.1186/s12906-026-05509-w)
Supplement: Supplementary file 4 [file 12906_2026_5509_MOESM4_ESM.docx]

**Supporting information 4.**

**Table S3. Comparison of outcome measures before and after intervention in two groups of children (mean ± SD / median [P25–P75]).**

|  | **Group** | **Indicator** | **Pre-Intervention** | **Post-Intervention** | **t/*Z*-value** | ***P*-value** |
| --- | --- | --- | --- | --- | --- | --- |
| **PP** | **Experimental (n=20)** | **MABC-2** | 56.75±9.87 | 69.15±9.63 | 11.619 | <0.001 |
|  |  | manual dexterity | 19.10±5.57 | 21.10±5.46 | 1.99 | 0.061 |
|  |  | aiming and catching | 11.50±3.35 | 15.75±2.94 | 5.396 | <0.001 |
|  |  | balance | 26.15±6.62 | 32.30±7.99 | 6.781 | <0.001 |
|  |  | **Static balance** |  |  |  |  |
|  |  | Sway area ^*^ | 150.00(103.00,278.75) | 114.50(84.00,135.00) | -2.128 | 0.033 |
|  |  | Sway path length | 431.80±142.05 | 278.75±92.42 | -4.791 | <0.001 |
|  |  | **Dynamic postural stability** |  |  |  |  |
|  |  | stability limit index | 65.62±15.25 | 71.55±11.39 | 3.322 | 0.004 |
|  | **Control (n=16)** | **MABC-2** | 54.00±13.04 | 55.56±14.06 | 1.501 | 0.154 |
|  |  | manual dexterity | 16.69±7.24 | 17.56±6.26 | 0.835 | 0.417 |
|  |  | aiming and catching | 12.75±4.07 | 13.44±2.63 | 0.902 | 0.381 |
|  |  | balance | 24.56±8.95 | 24.56±9.74 | 1.571 | 1.000 |
|  |  | **Static balance** |  |  |  |  |
|  |  | Sway area ^*^ | 254.00(175.50,306.75) | 249.50(182.00,329.00) | -0.569 | 0.569 |
|  |  | Sway path length **^*^** | 408.00(314.75,509.50) | 434.00(334.25,513.50) | -0.078 | 0.938 |
|  |  | **Dynamic postural stability** |  |  |  |  |
|  |  | stability limit index | 55.69±16.59 | 58.74±13.41 | 1.89 | 0.078 |
| **ITT** | **Experimental (n=20)** | **MABC-2** | 56.75±9.87 | 69.15±9.63 | 11.619 | <0.001 |
|  |  | manual dexterity | 19.10±5.57 | 21.10±5.46 | 1.99 | 0.061 |
|  |  | aiming and catching | 11.50±3.35 | 15.75±2.94 | 5.396 | <0.001 |
|  |  | balance | 26.15±6.62 | 32.30±7.99 | 6.781 | <0.001 |
|  |  | **Static balance ^*^** | 150.00(103.00,278.75) | 114.50(84.00,135.00) | -2.128 | 0.033 |
|  |  | Sway area | 431.80±142.05 | 278.75±92.42 | 4.791 | <0.001 |
|  |  | Sway path length **^*^** | 150.00(103,278.75) | 114.50(84.00,135.00) | -2.128 | 0.033 |
|  |  | **Dynamic postural stability** |  |  |  |  |
|  |  | stability limit index | 65.62±15.25 | 71.55±11.39 | 3.322 | 0.004 |
|  | **Control (n=20)** | **MABC-2** | 54.5±11.73 | 56.31±12.70 | 2.165 | 0.043 |
|  |  | manual dexterity | 16.8±6.45 | 17.69±5.60 | 1.059 | 0.303 |
|  |  | aiming and catching | 12.45±3.78 | 13.22±2.43 | 1.240 | 0.230 |
|  |  | balance | 25.25±8.16 | 25.05±8.76 | -0.308 | 0.762 |
|  |  | **Static balance** |  |  |  |  |
|  |  | Sway area ^*^ | 225.00(173.00,292.25) | 238.50(190.25,314.00) | 1.382 | 0.167 |
|  |  | Sway path length **^*^** | 409.50(339.50,498.50) | 419.00(340.00,490.00) | 0.093 | 0.926 |
|  |  | **Dynamic postural stability** |  |  |  |  |
|  |  | stability limit index | 57.27±15.33 | 60.32±12.63 | 2.378 | 0.028 |

Note: * Data are not normally distributed; the Mann-Whitney U test for two independent samples was used
