# Supplementary material for: Baduanjin improves motor function and cortical activation in children with developmental coordination disorder: a randomized controlled trial
Source: BMC Complement Med Ther. 2026 Aug 13;26:343. doi: 10.1186/s12906-026-05509-w (PMC13749755; doi:10.1186/s12906-026-05509-w)
Supplement: Supplementary file 5 [file 12906_2026_5509_MOESM5_ESM.docx]

**Supporting information 5.**

**Figure S3**.** Brain activation maps during the drawing trail task before and after intervention in the experimental Group.**
(Note: a. Brain regions showing significant activation differences before and after intervention; b. Brain activation map before intervention; c. Brain activation map after intervention.)

**Figure S4. Brain activation maps during the aiming and catching task before and after intervention in the experimental Group.**
(Note: a. Brain regions showing significant activation differences before and after intervention; b. Brain activation map before intervention; c. Brain activation map after intervention.)

**Figure S5. Brain activation maps during the one-leg balance task before and after intervention in the experimental Group.**
(Note: a. Brain regions showing significant activation differences before and after intervention; b. Brain activation map before intervention; c. Brain activation map after intervention.)

**Figure S6. Brain activation maps during the drawing trail task before and after intervention in the control Group.**
(Note: a. Brain regions showing significant activation differences before and after intervention; b. Brain activation map before intervention; c. Brain activation map after intervention.)

**Figure S7. Brain activation maps during the aiming and catching task before and after intervention in the control Group.**
(Note: a. Brain regions showing significant activation differences before and after intervention; b. Brain activation map before intervention; c. Brain activation map after intervention.)

**Figure S8. Brain activation maps during the** **one-leg balance task before and after intervention in the control Group.**
(Note: a. Brain regions showing significant activation differences before and after intervention; b. Brain activation map before intervention; c. Brain activation map after intervention.)
