# Supplementary material for: Baduanjin improves motor function and cortical activation in children with developmental coordination disorder: a randomized controlled trial
Source: BMC Complement Med Ther. 2026 Aug 13;26:343. doi: 10.1186/s12906-026-05509-w (PMC13749755; doi:10.1186/s12906-026-05509-w)
Supplement: Supplementary file 6 [file 12906_2026_5509_MOESM6_ESM.docx]

**Supporting information 6.**

### **Figure S9. Ethics approval document**.****

**Figure S10. Informed consent form.**
